# Supplementary material for: Analysis of rDNA reveals a high genetic diversity of Halophila major in the Wallacea region
Source: PLoS One. 2021 Oct 22;16(10):e0258956. doi: 10.1371/journal.pone.0258956 (PMC8535426; doi:10.1371/journal.pone.0258956)
Supplement: S1 Table — a: First identified as Halophila euphlebia. b: used in phylogenetic analysis, c: used in population genetic, d: used in time-calibrated phylogeny. (DOCX) [file pone.0258956.s001.docx]

| No | Species | GB | Countries | Regions | Sources |
| --- | --- | --- | --- | --- | --- |
| 1 | *Halophila major*^b,c^ | AB436927 | Thailand | Sunda Shelf  (I) | Uchimura et al. 2008 |
| 2 | *Halophila major*^b,c,d^ | KP408256 | Thailand |  | Tuntiprapas et al. 2015 |
| 3 | *Halophila major*^c^ | KP408257 | Thailand |  | -/- |
| 4 | *Halophila major*^c^ | KP408258 | Thailand |  | -/- |
| 5 | *Halophila major*^c^ | KP408259 | Thailand |  | -/- |
| 6 | *Halophila major*^c^ | KP408260 | Thailand |  | -/- |
| 7 | *Halophila major*^c^ | KP408261 | Thailand |  | -/- |
| 8 | *Halophila major*^c^ | KP408262 | Thailand |  | -/- |
| 9 | *Halophila major*^c^ | KP408263 | Thailand |  | -/- |
| 10 | *Halophila major*^c^ | KP408264 | Thailand |  | -/- |
| 11 | *Halophila major*^c^ | KP408265 | Thailand |  | -/- |
| 12 | *Halophila major*^b,c^ | KF620352 | Myanmar |  | Nguyen et al. 2014 |
| 13 | *Halophila major*^c^ | KC175910 | Viet Nam |  | Nguyen et al. 2013 |
| **14** | ***Halophila major*^c^** | **MW084376** | **Viet Nam** |  | **This present study** |
| **15** | ***Halophila major*^c,d^** | **MW084377** | **Viet Nam** |  | **This present study** |
| **16** | ***Halophila major*^c^** | **MW084375** | **Viet Nam** |  | **This present study** |
| 17 | *Halophila major*^b,c^ | KF620340 | Malaysia |  | Nguyen et al. 2014 |
| 18 | *Halophila major*^b,c^ | MT028356 | Indonesia | Wallacea  (II) | Kurniawan et al. 2020 |
| 19 | *Halophila major*^b,c,d^ | AB436928 | Indonesia |  | Uchimura et al. 2008 |
| 20 | *Halophila major*^b,c^ | AB436926 | Indonesia |  | -/- |
| 21 | *Halophila major*^b,c^ | MT028354 | Indonesia |  | Kurniawan et al. 2020 |
| 22 | *Halophila major*^b,c^ | MT028353 | Indonesia |  | -/- |
| 23 | *Halophila major*^b,c^ | MT028355 | Indonesia |  | -/- |
| 24 | *‘Halophila ovalis’*^b,c^ | AF366416 | Philippines |  | Waycott et al. 2002 |
| 25 | *‘Halophila australis’*^b,c^ | AF366414 | Australia | Sahul Shelf  (III) | Waycott et al. 2002 |
| 26 | *‘Halophila ovalis’*^c^ | AF366415 | Australia |  | -/- |
| 27-42 | *Halophila major*^b,d^ | MT347852-67 | Sri Lanka | Bay of Bengal (IV) | Liu et al. 2020 |
| 43-71 | *Halophila major*^b,d^ | MT347869-97 | Sri Lanka |  | -/- |
| 72 | *Halophila major*^b^ | MT347936 | Sri Lanka |  | -/- |
| 73 | *Halophila major*^a,b^ | AB243957 | Japan | Coast of Japan  (V) | Uchimura et al. 2006 |
| 74 | *Halophila major*^a,b^ | AB243968 | Japan |  | -/- |
| 75 | *Halophila major*^a.,b,d^ | AB243962 | Japan |  | -/- |
| 76 | *Halophila major*^a,b^ | AB243966 | Japan |  | -/- |
| 77 | *Halophila major*^a,b^ | AB243967 | Japan |  | -/- |
| 78 | *Halophila major*^a,b^ | AB243965 | Japan |  | -/- |
| 79 | *Halophila minor*^b,d^ | AF366406 | Philippines |  | -/- |
| 80 | *Halophila nipponica*^b,d^ | AB436931 | Japan |  | Uchimura et al. 2008 |
| 81 | *Halophila ovalis*^d^ | MF371443 | Egypt |  | Nguyen et al. 2018 |
| 82 | *Halophila ovalis*^b^ | AB243973 | Japan |  | Uchimura et al. 2006 |
| 83 | *Halophila ovalis*^d^ | AB436939 | Thailand |  | Uchimura et al. 2008 |
| 84 | *Halophila ovalis*^d^ | AB436940 | Indonesia |  | -/- |
| 85 | *Halophila ovalis*^d^ | AF366431 | Australia |  | Waycott et al. 2002 |
| 86 | *Halophila ovalis*^d^ | KC175909 | Viet Nam |  | Nguyen et al. 2013 |
| 87 | *Halophila ovalis*^b^ | KC175908 | Viet Nam |  | -/- |
| 88 | *Halophila* decipiens^b,d^ | KC175913 | Viet Nam |  | -/- |
| 89 | *Halophila stipulacea*^b,d^ | AF366436 | Italy |  | Waycott et al. 2002 |
| 90 | *Halophila tricostata*^d^ | AF366438 | Australia |  | -/- |
| 91 | *Halophila spinulosa*^d^ | AF366440 | Malaysia |  | -/- |
| 92 | *Halophila engelmannii*^d^ | AF366404 | USA |  | -/- |
| 93 | *Halophila beccarii*^b,d^ | AF366441 | Viet Nam |  | -/- |
