# Supplementary material for: Analysis of rDNA reveals a high genetic diversity of Halophila major in the Wallacea region
Source: PLoS One. 2021 Oct 22;16(10):e0258956. doi: 10.1371/journal.pone.0258956 (PMC8535426; doi:10.1371/journal.pone.0258956)
Supplement: Attachment — Submitted filename: responses to Reviewers.docx [file pone.0258956.s002.docx]

Mr. Xuan-Vy Nguyen
Institute of Oceanography, Viet Nam Academy of Science and Technology
01 Cau Da, Nha Trang City, Viet Nam

February 7^th^, 2021

Dear Editor, dear reviewers

Thank you very much for your detailed suggestions and comments. All your suggestions are very helpful for us to improve the manuscript. All changes are highlighted in the newest version. Our responses are below:

List of revisions

| **Recommendation** | **Responses** |
| --- | --- |
| **Editor** | |
| Q1. Please ensure that your manuscript meets PLOS ONE's style requirements, including those for file naming | R1: Thank you very much, the new version was formatted following guidelines of journal including the title, authors, affiliations, figure, table and support information. |
| Q2. In your Methods section, please provide additional location information of the sampling sites, including geographic coordinates for the data set if available. | R2: We mentioned the additional location information of the sampling sites, including geographic coordinates for the data set in the method section. |
| Q3. In your Methods section, please provide additional information regarding the permits you obtained for the work. Please ensure you have included the full name of the authority that approved the sampling sites access and, if no permits were required, a brief statement explaining why. | R3: We added the additional information regarding the permits our team obtained for the work at three locations |
| Q4. Please remove any funding-related text from the manuscript and let us know how you would like to update your Funding Statement. Currently, your Funding Statement reads as follows  Please include the updated Competing Interests Statement and Funding Statement in your cover letter. We will change the online submission form on your behalf | R4. Yes, we removed funding-related text from the manuscript. The cover letter is updated. |
| Q5. We note that Figures 1 and 4 in your submission contain map images which may be copyrighted. All PLOS content is published under the Creative Commons Attribution License (CC BY 4.0), which means that the manuscript, images, and Supporting Information files will be freely available online, and any third party is permitted to access, download, copy, distribute, and use these materials in any way, even commercially, with proper attribution. For these reasons, we cannot publish previously copyrighted maps or satellite images created using proprietary data, such as Google software (Google Maps, Street View, and Earth). For more information, see our copyright guidelines: http://journals.plos.org/plosone/s/licenses-and-copyright. | R5. The background map used in the manuscript is from NOAA/NGDC. For our previous papers, we contacted the NOAA/NGDC for permission. They answered to us that it is public domain and available for use. Therefore, we wrote “Source of digital map: The National Oceanic and Atmospheric Administration (NOAA), USA, public domain data”  For Fig. 4. The map was integrated in the PopArt software that was used to produce Fig. 4. To be safe, we modified Fig. 4 with background map used in Figure 1. |
| Q6. Please include a caption for figures 5 and 6. | R5. Captions for figures 5 and 6 were added |
| Q7. Please include captions for your Supporting Information files at the end of your manuscript, and update any in-text citations to match accordingly. Please see our Supporting Information guidelines for more information | R6. Caption for Supporting Information file was included at the end of your manuscript.  We updated citations to match accordingly. |
| **Reviewer 1** | |
| Q1. There are several scientific names of species that are not italicized and according to the standard of writing, and should only be the scientific name of the species in italics. And then, there is a lot of writing that needs to be improved, especially related to continuity between sentences and between paragraphs, as well as table and unit. The units should use symbols that have been defined internationally, and are consistent. | R1. Thank you very much, the manuscript was improved. Please see the changes that were highlighted |
| Q2. Typos “fom” | R2. Thank you very much, we corrected |
| Q3. When biological species that is the first time mentioned in the text, it should attach the authority name and year | R3. We added the authority name and year after scientific name |
| Q4. Page 6: Phylogenetic analysis section….”, including three sequences obtained in this study and 73 sequences of known Halophila sections retrieved from GenBank..”. Total 76 but in Result All sequences were 78 | R4. We changed to …”73 sequences of known *Halophila* species” 69 ITS sequences of *H. major*. Please see the S1 Table, |
| Q5. Page 7: “Morphological observation and phylogenetic analysis of Halophila major in Vietnamese waters”. Suggestion to reduce, “Halophila major in Vietnamese waters.” | R5. Thank you very much, we agree to reduce |
| Q6. Page 8: “A total of 78 ITS sequences (including three new sequences from the present study) of H. major collected in five geographic areas: Sunda Shelf (I), Wallacea (II), Sahul Shelf (III), Bay of Bengal (IV) and coast of Japan (V)”.Total sequences are 78 or 76?; this sentence prefer in phylogenetic analysis in method. | R6. Yes, we checked and corrected the numbers, please see my answer at R4. |
| Q7. Page 9: in Table 3, please consistent, areas or region is used based on the context | R7. Thank you for your finding, we changed all “area” to “region” except where area was used for specific locations in Viet Nam |
| Q8. Page 10: First paragraph “Area” similar comment above. | R8. Please, see our answer in R7 |
| Q9. Page 11: The number of branching veins for H. ovalis 3-4 from Indonesia samples. Generally, branching cross veins of H. ovalis is 3-4 pairs. How about the branching cross veins of H. ovalis in Vietnam? | R9. Thank you very much for your sharing, it is the same, 3-4 from the Vietnamese samples |
| Q10. Page 11: “….whereas samples collected in Malaysia, Indonesia and Thailand tend to form a single clade”. Insert Fig. 3 and Why it placed in single clade, How it related with this finding? | R10. We inserted Fig 3. The group IV contains materials from Malaysia, Indonesia and Thailand were performed by data. There are several articles mentioning long‐distance dispersal, positively buoyant shoots with attached rhizomes or seedlings having high potential for long‐distance dispersal. Therefore, DNA fingerprinting should be applied to investigate the population structure and gene flows among populations of *Halophila ovalis* or *Halophila major* from SEA in further research studies. |
| Q11. Page 12: Typo. “…..in regions II (Sunda Shelf) and III (Wallacea)”. it means Sunda Shelf (region I) and Wallacea (II) | R11. Thank you very much, we corrected |
| Q12. Improve the quality of all Figure (should be min. 350 DPI) follow the journal requirement. | R12. We agree, all figures were improved |
| Q13. Figure 1. Suggestion: prefer polygon with different color or pattern to differentiate the region. | R13. Thank you for your suggestion. We tried polygon, but the land is polygon too. However, Figure 1 was modified following reviewer 2 by using different symbols coding different regions |
| Q14. Figure 2. Please insert the scale like 1 cm or 1 mm | R14. We agree, we edited the figure 2, the scale is 1 mm that was mentioned in figure caption. |
| **Reviewer 2** | |
| Q1: the title is not falling into their main conclusions (findings), because throughout the manuscript, they only discussed about the high genetic diversity in Wallacea with only few sentences in page 12. | R1. We add more sentences, it may support to the tittle, please see it in the highlight. |
| Q2. Second, they seem to be misunderstood the the definition of the term "monophyletic", based on their phylogenetic tree, they mentioned there are 8 subclades in H. major and form nonmonophyletic groups. However, since the sub clades the authors defined in this studied are not well supported at the tips. They is "no" subclade in H. major, and sequences of H. major around the world formed" a "monophyletic clade with well support (supporting values: 98/1). This is also supported by the TCS haplotype network which showed no sign of population partition. | R2. Thank you very much for your suggestion, we changed to smaller groups, and statement “and sequences of H. major around the world formed" a "monophyletic clade” |
| Q3. Third, they use same symbol to code geographic regions as well as 8 clades (I, II, III.....), which is really confusing while reading | R3. We changed different symbols to different regions in Fig. 1. Eight small groups were also modified |
| Q4. the resolution of the figures are generally low especially Figure 6. | R4. Thank you, we improved the resolution all figure |
| Q5. Page 5, spell out "DESS" | R5. We explained DESS solution. It is 20% dimethyl sulfoxide, 0.25 M disodium EDTA, and saturated NaCl. |
| Q6 Figure 1 caption: "where samples were collected change" to "where sequences were obtained from GenBank" | R6. Thank you very much. We changed, and modified the Figure 1 caption. Please see our answer R13 to reviewer 1. |
| Q7. "World type" "Red Sea type", you need to explain what are they or simply state previously been defined as "World type"and "Red Sea type" by someone.... There are many sentences developed from the erroneous statement "8 subclades", so the authors may need to reframe the manuscript based on the correct interpretations. | R7. We corrected and removed Red Sea type, simply we changed to “samples collected from Red Sea”, and we changed “subclade” to “group” |
| **Reviewer 3** | |
| Q1. The species Halophila major (H. euphlebia) was ‘re-established’ in 2006, therefore, using the prior to 2006 data from the geneBank to treat as “H. major” could be questionable. For example, the Halophila specimens from Shoalwater Bay, Australia have distinct linear leaf blades with L/W ratio 7-10; cross veins 8-12 (-14), unbranched…Based on the above morphological description, the specimens may not belong to H. major. | R1. Thank you very much, we agree with you. It is not easy to identify the *Halophila major* and closely related species based on morphological observation only. In this present study, first we used the sequence of *H. major* (Uchimura et al. 2008) for blasting in NCBI. All sequences with similarity more than 99% were used for the analysis. This group contained two sequences from Australia. Intra-species variation within *H. nipponica* were reported in Japan or *H. ovalis* from Malaysia. |
| Q2. Abstract stated: “H major was misidentified as H. ovalis in Viet Nam”. However, this statement did not appear in other sections of this manuscript nor any reference was given. | R2. The Halophila ovalis/major complex was reported from Viet Nam in 2013 (Ref 13), and H. major was only found at one off-shore island. Therefore, we are now checking the Halophila ovalis/major complex from three different locations. |
| Q3. Introduction: References [4, 5] did not use section Halophila. Furthermore, H. australis, H. capricorni, and H. sulawesii also include in this section. However, the latest Halophila taxonomy review shows that section Halophila does not include these three species and also not H. stipulacea and H. decipiens (see Kuo 2020). | R3. We modified these sentences, 13 species in section *Halophila* was updated, and the Ref. Kuo, 2020 was updated. Please see our answer to reviewer 1. |
| Q4. Fig. 3 H. major has eight subclasses. Those from Japan belong to Class III and VIII; Australia belongs to III and VI; while Thailand had IV and VII. Why two different subclasses in the same country (Japan, Australia, Thailand)? Any morphological or environmental difference of the specimens in two different subclasses from the same country? | R4. It is a really nice question. Your question is something that is related to Q11 from reviewer 1. In Australia, I think that the geographic distance between two sites from Australia, it may be about 3,000 km from the west side to the east site. In the case of Japan, one site is Okinawa where the water temperature is warmer than the mainland (the other site), but we cannot explain in the case of Thailand. Two haplotypes were found in the Andaman Sea. Hopefully, we will collect more materials from above locations, then we use SSRs approach to estimate the migration among populations in SEA and the other regions |
| Q5. References: should list: Liu et al. (2020) Genetic identification and hybridizationin the seagrass genus Halophila (Hydrocharitaceae) in Sri Lanka waters. PeerJ 2020, 8, e10027 | R5. Thank you, we cited and added this Ref. |
| Q6. Authors estimated relative divergence of H. major and H. ovalis were 6.64 Mya, that was much older than Kim et al.’s estimation of 3.4 Mya. The authors of this manuscript explained these differences were due to ‘technical aspect’ and the length of sequences in the dataset used. They should explain what is ‘technical aspect’. | R6. Thank you for your interesting comment. The different estimated relative divergence in Kim et al. 2017 can be explained as follows: Figure 4A (estimated relative divergence = 3.5 Mya for H. ovalis), and Figure 4B (estimated relative divergence is about 8 Mya for H. ovalis clade). The software applied in their work was NETWORK 4.6 program. Therefore. the 95% Highest Density Probability (HPD) intervals and posterior probability values were not included. In this present study we used the popular software for estimating relative divergences Beast v2.5 |
| Q7 Authors estimated the divergence between section Halophila and section Microhalophila was 32.18 Mya, this estimation implies that the divergence of the genus Halophila would be much earlier than that estimated divergence of genus Halophila in the family Hydrocharitaceae was only 19.41 Mya by Chen et al. The authors of this manuscript should discuss these vast differences. | R7. We agree with you. Chen et al used multilocus in plastid DNA while this present study used rDNA. Unfortunately, there are only limited data of plastid DNA from members of *Halophila*. |

We hopefully fulfilled all points raised.

Sincerely Yours

Xuan-Vy Nguyen
