# Supplementary material for: Analysis of rDNA reveals a high genetic diversity of Halophila major in the Wallacea region
Source: PLoS One. 2021 Oct 22;16(10):e0258956. doi: 10.1371/journal.pone.0258956 (PMC8535426; doi:10.1371/journal.pone.0258956)
Supplement: Attachment — Submitted filename: responses to Reviewers.docx [file pone.0258956.s003.docx]

Dr. Xuan-Vy Nguyen
Institute of Oceanography, Viet Nam Academy of Science and Technology
01 Cau Da, Nha Trang City, Viet Nam

April 25^th^, 2021

Dear Editor

Thank you very much for forwarding the detailed suggestions and comments from the reviewers. All suggestions have been very helpful for us to improve the manuscript. All changes are highlighted in the newest version. Our responses are below:

List of revisions

| **Recommendation** | **Responses** |
| --- | --- |
| **Editor** | |
| Q1. Note that one of the reviewers still expects a few changes before his manuscript is accepted for publication. Please make these adjustments so that I can proceed with the revision of the manuscript. | R1: Thank you very much, please see our responses to the reviewers below. |
| **Reviewer 1** | |
| Q1. In the discussion section, suggestions should be made regarding the use of fresh specimens and adding locations, and things that become input and challenges in the future, including collaborative research in order to really get more complete data to better find out its genetic diversity. | R1. Thank you very much for your suggestions, we add more information in discussion part (yellow highlight). |
| Q2. There is something that needs to be ascertained from the phylogenetic tree, is H. major in Australia really any specimens based on morphological evidence? If there is, it may be stated that the source of the citation is that H. major is found in Australia in Materials and methods. | R2. We agree with you, “*H. major”* was previously treated as *H. ovalis* by Waycott et al. (2002). Uchimura et al. (2008) suggested that it should be *H. major*. Therefore, we add Uchimura et al. (2008) and Waycott et al. (2002) when we mention that *H. major* occurs in Australia (yellow highlight. Line 63, page 3) |
| **Reviewer 2** | |
| Q1: Although authors have replied most of my comments, there is one exception which related to the symbol (I, II, III etc.) they used in the Figure 1 and in the phylogenetic tree to represent geographic areas and "groups", respectively. | R1. Thank you very much for your finding the conflicts between Fig. 1 and Fig. 3. We kept I, II, III in Fig. 1. We changed 1,2,3… in Figure 3 |
| Q2 They are several things that I concern while reviewing this revision. Here I list them point by point as follows. Line 75-76: Delete "In addition, the study....on the IST marker." Line 88-89: Unclear, had to rephrase. Line 106-108: This sentence is odd, need to rewrite to clarify what you were trying to say. Line 109-110:How the genetic partition occur "within" and "among" barrier ? The authors may need to clarify this or simply make this sentence more straight forward. Line 129: Use "sequences of other regions" instead of "other regions sequences" Line 140: Use "the" instead of "this". | R2. Thank you for your recommendation  Line 75-76: Deleted  Line 88-89: Yes, we modified the sentence.  Line 106-108: Thank you, we rewrote the sentence.  Line 109-110: We changed this sentence.  Line 129: Yes, we modified.  Line 140: done |
| Q3. Line 243-244: I would like the authors to provide the supporting values of each groups they defined. Because technically, if those groups are not support by supporting values they should not be defined as different group. I know the authors already used group instead of clade. One thing the authors can do is be honest with what they found (groups are not well-supported) and add up the information that the grouping of those haplotypes might affiliate with geographic location based on the haplotype network instead of define them as groups without substantial evidence. | R3. Thank you, we agree with you, we added the supporting values to the tree. Therefore, the Fig. 3 was modified. We agree with you, *H. major* is a monophyletic group, but the trend seems to separate it into groups with low supporting values (not for all groups). You can follow our changes by highlighting them. For the haplotype network, we also agree with you and modified Fig. 5. We removed group A,B. The text was also modified, please see the highlighted passages. |
| Q4. Line 328-329: The authors should explain what is the "similar way" they mentioned here? Because the original sentence is not very clear. Additionally, in the tree generated by Waycott et al. 2002, those groups found in H. ovalis samples were not well-supported, therefore, they also over-interpreted their data almost 20 years ago. And I truly believe we should not follow the conclusion which is not correct. | R4. We removed two last sentences of this paragraph. |
| Q5. Line 382-383: You only have the morphological data from Viet Nam, therefore, I don't think you can make this conclusion while you mentioned a broad area in previous sentence. | R5. We changed to monophyletic group that was not changed in the previous version. |
| Q6 I strongly recommend the authors send their next revision for English editing before submitting back to PloS One, because I did not correct them across the manuscript. I am not a native speaker too, and I always send my manuscript for English editing before submitting, just because it actually help the audiences to read and receive the information that we are trying to deliver. | R6. Thank you for your recommendation, we will consider your suggestion. Our co-authors, especially Prof. Lawrence M. Liao and Prof. J. Papenbrock carefully checked and edited the manuscript in the final version. |

We hopefully fulfilled all points raised.

Sincerely yours,

Xuan-Vy Nguyen
