# Supplementary material for: Analysis of rDNA reveals a high genetic diversity of Halophila major in the Wallacea region
Source: PLoS One. 2021 Oct 22;16(10):e0258956. doi: 10.1371/journal.pone.0258956 (PMC8535426; doi:10.1371/journal.pone.0258956)
Supplement: Attachment — Submitted filename: Response to Reviewer.docx [file pone.0258956.s004.docx]

Response to Reviewers

| **Recommendation** | **Responses** |
| --- | --- |
| **Editor** | |
| Q1. Principally, results as presented do not clearly support the primary conclusion of significant genetic isolation of *Halophila major* within the central Indo-Pacific (lines 42-43 and elsewhere) | R1: We agree with you, we changed to non-significant genetic isolation. |
| Q2. The lack of support for phylogenetic divergence within H. major is acknowledged in the text (eg. lines 381-383) yet elsewhere support for the putative groupings is implied (eg. lines 38-39). At a minimum, this confuses the discussion and should be clarified. | R2: We modified lines 38-39, and line 381-383 |
| Q3. As described in Excoffier et al. 2010, AMOVA allows the hierarchical partitioning of genetic variation among populations (phiSC), among regions (phiCT), and among individuals within populations (phiST). As presented, table 4 indicates a non-significant phiCT. This may simply be a typo, as suggested by the lack of haplotype sharing among sites (table 2). If, however, this is not a typo, I feel the primary conclusions of the manuscript are unsupported and significant revisions would be needed before publication. | R3. We agree with you, it is typos, Table 4 was corrected |
| Q4. I am therefore recommending major revisions with the caveat that my concerns about project findings may be addressed if AMOVA results in table 4 are not correct as presented. | R5. We changed the results of Table 4. Non-significant differences were found among regions, but significant differences were presented among populations |
| Q5. Lines 38-39: Misidentification of Viet Nam H. major was described in a prior study and should not be highlighted here as a new finding | R.5 It was removed |
| Q6. Line 40: It is unclear who “they” is referencing. Viet Nam samples only? | R6. We modified the sentence. |
| Q7. Lines 42-43: If the AMOVA table is correct as presented this conclusion will need to be revised | R7. Please see our responses in R5 and R6 |
| Q8. Lines 54-59: Author taxonomy citations are inconsistent. My understanding is that presenting the authors last name is standard format. Please confirm and correct as needed. | R8. We used authors last name, and they are consistent now |
| Q9. Line 104-105: Sentence starting with “Oceanic currents…” is unclear. Please revise. | R9. We used “sea currents” |
| Q10. Line 118: Delete “area” | R10. Done |
| Q11. Line 146: Change “Information of the samples…” to “Sample information…” | R11. Done |
| Q12. Methods: Significant methodological details are missing…How were pairwise nucleotide differences estimated? What mutational model was used? What is a p-distance? How were AMOVA groupings determined and what were they? Highest Probability Density (BEAST?) is not described. | R12. We added significant methodological details. |
| Q13. Lines 231-232: The described H. major groupings are unsupported and should be collapsed into a single branch. Alternatively, the text should be clarified here and elsewhere to avoid any suggestion that there is phylogenetic support for the groups. This does not alone refute the project findings, but it does add some uncertainty. | R13. Thank you very much, our changes to mention that the *H. major* groupings are unsupported. |
| Q14. Table 3: Type “summared” | Done |
| Q15. Lines 281-282: The AMOVA statement will need to be revised if table 4 is correct. |  |
| Q16. Line 309: Typo “highest density probability” | Done |

We hopefully fulfilled all points raised.

Sincerely Yours

Xuan-Vy Nguyen
