# Supplementary material for: Analysis of rDNA reveals a high genetic diversity of Halophila major in the Wallacea region
Source: PLoS One. 2021 Oct 22;16(10):e0258956. doi: 10.1371/journal.pone.0258956 (PMC8535426; doi:10.1371/journal.pone.0258956)
Supplement: Attachment — Submitted filename: responses to Editor.docx [file pone.0258956.s005.docx]

Mr. Xuan-Vy Nguyen
Institute of Oceanography, Viet Nam Academy of Science and Technology
01 Cau Da, Nha Trang City, Viet Nam

August 24^th^, 2021

Dear Editor

Thank you very much for your detailed suggestions and comments. All your suggestions are very helpful for us to improve the manuscript. All changes are highlighted in the newest version. Our responses are below:

List of revisions

| **Recommendation** | **Responses** |
| --- | --- |
| **Editor** | |
| Q1. Lines 37-38 "show variation" is unclear. For clarity I recommend indicating among what groups variation was observed. With the addition of this information I as well recommend splitting this statement into two sentences, the first highlighting where variation is observed and the second indicating low support for regional H. major groupings | R1: We agree with you, we changed by splitting into two sentences. |
| Q2. Lines 105-106.. perhaps something like "Oceanic currents can act to both promote or limit gene-exchange" would be more clear. | R2: Thank you for your suggestion. We changed |
| Q3. Figure 1: The Myanmar  site currently shows a solid circle but instead should be a solid diamond based on the figure 1 legend. | R3. I think it should by a solid circle, please see our highlight on the figure 1 legend |
| Q4. Line 146: "Sample" not "Samples" | R4. Done |
| Q5. Lines 191-193: Suggested revision "The average number of nucleotide differences between sampling locations for the full ITS fragment and per nucleotide was estimated in Mega X (40) using the Kimura 2-parameter model (39)." | R.5 Thank you for your correction, we changes. We also changed in the Reference. Please see our highlight |
| Q6. Lines 249-250: Which ML and base values are you referring to? I would not consider the values in general to be high given the lack of support for the H. major group. Please revise to avoid giving the false impression that there is significant support for H. major groupings. | R7. We removed the sentence. |
| Q8. Line 251 and elsewhere: Table 2 presents two different estimates of 'evolutionary divergence', one for the full fragment and one standardized estimate of the per nucleotide rate. I recommend changing how these measures are referred to here and elsewhere for clarity. Perhaps something like "Evolutionary divergence as measured by estimated total fragment and per nucleotide differences...". | R8. We changed |
| Q9. Table 2 caption: See my note above.. | R9. We changed |
| Q10. Table 3 caption: "Summarized statistics of haplotypes" is unclear. I recommend something like "Summarized Halophila major sample size, number of haplotypes observed, and estimates of genetic diversity | R10. Done |
| Q11. Table 3: I only just noticed that you've included estimates of Tajima's D and Fu's Fs here but they are not mentioned in the results nor discussion. I recommend deleting these measures from table 3 and the methods unless you can find a meaningful way to include the results | R11. We removed in both Table and in Method |
| Q12. Line 401: Recommend changing "but it trends to form..." to "with unique haplotypes occurring among regions but no or low support for regional groupings." | R12. Thank you, we changed |

We hopefully fulfilled all points raised.

Sincerely Yours

Xuan-Vy Nguyen
